# Autophagy in Traumatic Brain Injury: A New Target for Therapeutic Intervention

**DOI:** 10.3389/fnmol.2018.00190

**Published:** 2018-06-05

**Authors:** Li Zhang, Handong Wang

**Affiliations:** Department of Neurosurgery, Jinling Hospital, School of Medicine, Nanjing University, Nanjing, China

**Keywords:** traumatic brain injury, autophagy, methods, molecular mechanisms, pharmacological modulation

## Abstract

Traumatic brain injury (TBI) is one of the most devastating forms of brain injury. Many pathological mechanisms such as oxidative stress, apoptosis and inflammation all contribute to the secondary brain damage and poor outcomes of TBI. Current therapies are often ineffective and poorly tolerated, which drive the explore of new therapeutic targets for TBI. Autophagy is a highly conserved intracellular mechanism during evolution. It plays an important role in elimination abnormal intracellular proteins or organelles to maintain cell stability. Besides, autophagy has been researched in various models including TBI. Previous studies have deciphered that regulation of autophagy by different molecules and pathways could exhibit anti-oxidative stress, anti-apoptosis and anti-inflammation effects in TBI. Hence, autophagy is a promising target for further therapeutic development in TBI. The present review provides an overview of current knowledge about the mechanism of autophagy, the frequently used methods to monitor autophagy, the functions of autophagy in TBI as well as its potential molecular mechanisms based on the pharmacological regulation of autophagy.

## Introduction

Traumatic brain injury (TBI) is one of the leading causes of disability and death in modern society, resulting in high medical costs (Brooks et al., 2013). It is defined as any head injury with traumatic etiology, such as penetrating or blunt trauma and non-accidental injury. The pathological process of TBI includes both primary and secondary brain injury. Although the primary brain damage is the major factor determining the patients’ outcomes, the secondary brain damage induced by multiple pathological processes, such as inflammation, cell death, apoptosis, oxidative stress and impaired calcium and iron homeostasis, provides the possibility for clinical intervention (Zhang and Wang, 2018). Despite the efforts on searching effective methods to attenuate the secondary brain injury, patients suffering with TBI always end up with poor prognosis (Sun et al., 2015). Therefore, new and effective strategies of treatment are urgently needed to reduce the heavy disease and economic burden.

Autophagy is a self-catabolic process by which cells conserve and recycle their organelles in a stressed or nutrient-deprived state (Levine and Kroemer, 2008). This process is essential to maintain the metabolism essential for cell survival under stress situations. However, dysfunction of autophagy is involved in multiple diseases, including infectious diseases, cancers and TBI (Lipinski et al., 2015; Byun et al., 2017). Therefore, clarifying the molecular mechanisms of this degradation process may contribute to develop novel treatment protocols for therapeutic purposes. In the present study, we summarize the process of autophagy and its role in TBI as well as the associated molecular mechanisms and its regulated agents.

## The Process of Autophagy

**Figure 1:**
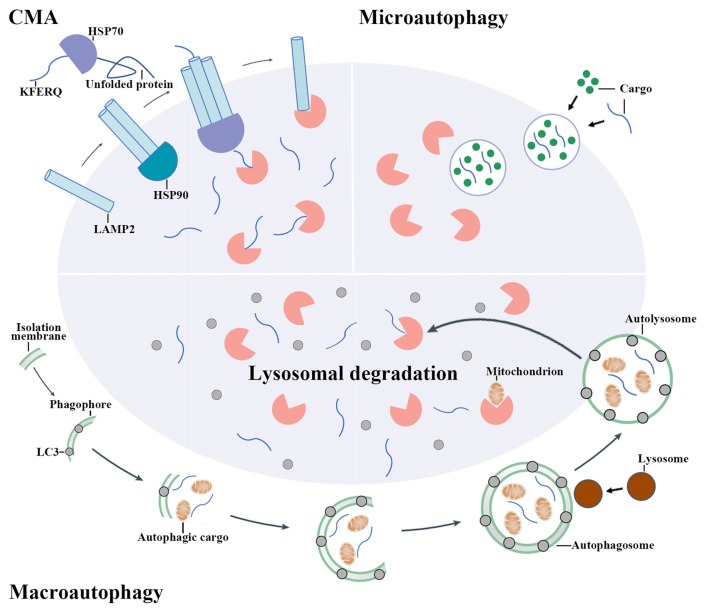
The mechanisms of autophagy pathway. There are three main types of autophagy including chaperon-mediated autophagy (CMA), microautophagy and macroautophagy. CMA involves the recognition of autophagic cargoes bearing a Lys-Phe-Glu-Arg-Gln (KFERQ) motif by heat shock proteins (HSPs), which is followed by the lysosomal-associated membrane protein 2 (LAMP2)-dependent translocation of chaperoned autophagic cargoes across the lysosomal membrane. By contrast, cargo delivery during microautophagy occurs upon the direct invagination of the lysosomal membrane. During macroautophagy, an isolation membrane encloses a portion of cytoplasm, forming a characteristic double-membraned organelle named autophagosome. Autophagosome then fuses with lysosome to form autolysosome and the cytoplasmic components are subsequently degraded by lysosomal enzymes.

Autophagy is a process that degrades cytoplasmic proteins and organelles. Generally, there are three types of autophagy that have been proposed up to now, including chaperon-mediated autophagy (CMA), microautophagy and macroautophagy. They distinguish from each other by how autophagic substrates are delivered to lysosome (Figure 1; Kaur and Debnath, 2015). CMA is only observed in mammalian cells. In CMA, autophagic substrates are directly translocated across the lysosomal membrane dependent on the Lys-Phe-Glu-Arg-Gln (KFERQ) motif, cytosolic chaperones of the heat-shock protein family and lysosomal-associated membrane protein 2 (LAMP2; Cuervo and Wong, 2014). Conversely, the delivery of autophagic substrates in microautophagy involves the direct invagination of lysosomal membrane (Mijaljica et al., 2011).

Macroautophagy (hereafter simply referred to “autophagy”) is the most widely studied and best known type among these three pathways. When autophagy is activated, the cytoplasmic proteins or organelles are enclosed by an isolation membrane to form autophagosome. Autophagosome then fuses with lysosome to form autolysosome and the cytoplasmic proteins or organelles are subsequently degraded by lysosomal enzymes (Mizushima and Komatsu, 2011).

## Methods for Monitoring Autophagy

Numerous reports have demonstrated that autophagy was activated in the brain after TBI. Accordingly, to exactly explore the role of autophagy in TBI, accurate methods to detect autophagy should be used. In this section, we summarize the frequently-used methods for detecting autophagy in TBI.

### Transmission Electron Microscope (TEM)

Transmission electron microscope (TEM) has long been considered as the “gold standard” for identification of autophagic vesicles. It is one of the most precise methods to examine autophagy, which provides the “seeing is believing” data (Murakawa et al., 2015). Under TEM, autophagosome can be typically identified as a double membrane containing cytoplasmic materials. The cytoplasmic materials in autophagosome include various organelles, such as endoplasmic reticulum (ER) and mitochondrion (Ylä-Anttila et al., 2009). When autophagosome fuses with lysosomal vesicle to form autolysosome, the outer membrane of autophagosome fuses with the lysosome membrane. The cytoplasmic organelles, still surrounded by the inner membrane, are delivered to the lysosome lumen. This inner membrane of autophagosome is then degraded to allow the degradation of the materials. Therefore, at the stage of autolysosome, TEM usually observes a monolayer structure containing numerous cytoplasmic materials (Eskelinen, 2008).

There are also limitations of TEM. For example, instead of whole cell, ultrathin slices of cell, usually of 70–80 nm thickness, could be observed. Therefore, the sample size is very small and it is difficult to get a concept of the size and total volume of different compartments inside the cell (Ylä-Anttila et al., 2009). Besides, TEM requires expensive equipment and professional technology, and is also time spending.

### Western Blot Assays of Autophagosome Marker Proteins

Beclin-1 is the mammalian ortholog of the yeast Apg6/Vps30 gene. It can promote the formation of autophagosome when overexpressed in mammalian cells (Liang et al., 1999). In addition, there is a lot of autophagy-related (ATG) proteins that mediate the activation of autophagy (Arroyo et al., 2014). Among them, the microtubule-associated protein light chain 3 (LC3) is widely used as a key marker for detection of autophagosome (Mizushima et al., 2010). LC3 is primarily synthesized in an unprocessed form, proLC3. When autophagy in activated, proLC3 is cleaved by ATG4 to form LC3-I, LC3-I then binds to phosphatidylethanolamine (PE) to become lipidated LC3 (LC3-II). Subsequently, LC3-II conjugates to both inner and outer membrane of autophagosome and contributes to the formation of autophagy. This process requires an ubiquitination-like reaction mediated by ATG3 and ATG7 (Kabeya et al., 2000). Therefore, the protein levels of Beclin-1, LC3, ATG3 and ATG7 detected by western blot are usually used to detect the formation of autophagy.

### Fluorescence Microscopy

LC3-I distributes in the cell uniformly when autophagy levels are low. However, upon the induction of autophagy, LC3-I turns to LC3-II and binds to the membrane of autophagosome, which can be visualized and quantified by fluorescence microscopy through counting LC3 puncta (Dolman et al., 2013). Monitoring LC3 puncta can depend on either the signal of green fluorescent protein (GFP) tagged to LC3 or immunofluorescence using an anti-LC3 antibody (Yoshii and Mizushima, 2017). The main limitation of fluorescence microscopy is that although LC3 puncta reflects an image of ATG structures in a cell, it does not illustrate that the autophagosome would reach the final stage of degradation.

### Autophagic Flux

Autophagy is initiated by the formation of autophagosome. Subsequently, autophagosome fuses with lysosome to form autolysosome and promotes cytoplasmic organoids degradation. This dynamic degradation process is named autophagy flux. There are generally three methods to detect autophagic flux, including LC3 turnover, autophagic substrate p62 degradation and tandem fluorescent-tagged LC3 (tfLC3) assay. At the stage of autophagic flux, the LC3-II is degraded by autolysosome. Thus, the lysosomal degradation of LC3-II reflects the progression of autophagic flux and assaying the expression of LC3-II in the presence of lysosomal inhibitors provides a reasonable way to monitor autophagic flux (Jiang and Mizushima, 2015). Moreover, p62, the adapter protein, promotes the ubiquitination of cytoplasmic organoids to autophagosome and degraded by autolysosome. Thus, the down-regulation of p62 suggests an occurrence of autophagic flux (Klionsky et al., 2016). In addition, a new autophagic flux observation method has been proposed by using a tandem monomeric red fluorescent protein (mRFP)-GFP-tfLC3. The GFP fluorescence signal is quenched in lysosome with an acidic compartment, whereas mRFP fluorescence signal remains its intensity in lysosome. Therefore, colocalization of GFP and mRFP fluorescence signal (yellow puncta) demonstrates that the tandem protein exists in an organelle which has not fused with lysosome, for example the autophagosome. Conversely, a single mRFP fluorescence signal without GFP (red puncta) demonstrates the translocation of tfLC3 to lysosome, that is, the formation of autolysosome. This novel system using tfLC3 allows a direct assessment of both autophagy induction and autophagy flux in the absence of any toxic inhibitors (Kimura et al., 2007).

## The Dual Role of Autophagy in TBI

Autophagy was firstly reported to be activated after TBI by Diskin et al. (2005). They found that in a mouse modified weight-drop TBI model, Beclin-1 was significant up-regulated at 4 h and 24 h in the cortical site of injured brain post-TBI. Double staining of Beclin-1 and TUNEL indicated that most of the injured cells exhibited double staining. Subsequently, a great number of studies have demonstrated that autophagy was induced after TBI. For example, in a mouse weight-drop TBI model, the expression of Beclin-1 was increased at 1 h post-TBI and peaked at 6 h while the expression of LC3-II was up-regulated shortly after TBI and peaked at 48 h in the injured cortex and hippocampus. Instead, the expression of p62 was decreased at 24 and 48 h in the injured cortex and hippocampus after TBI (Luo et al., 2011). Moreover, in a rat moderate fluid percussion TBI model, both autophagosome and autolysosome were detected in the injured cortical neurons from 4 h to 15 days after TBI under TEM and the expression of LC3-II was also up-regulated from 4 h to 15 days post-TBI in the injured cortex (Liu et al., 2008). More importantly, the induction of autophagy after TBI was not only found in animal models but also confirmed in clinical trials. Clark et al. (2008) reported that both LC3-II and Beclin-1 were increased in the injured temporal lobe cortex in patients suffering TBI. Although autophagy enhancement after TBI has been found in both animal and human models, the role of autophagy in TBI was still controversial. Different studies may draw different or even opposite conclusions.

### The Protective Role of Autophagy in TBI

The protective role of autophagy in TBI was firstly proposed by Erlich et al. (2007) using rapamycin. Rapamycin activate autophagy by inhibition of the phosphatidylinositide 3-kinases (PI3K)/protein kinase B (AKT)/mammalian target of rapamycin (mTOR) signaling pathway (Heras-Sandoval et al., 2014). They found that rapamycin could increase the protein levels of Beclin-1 and induce an augmented autophagic response after TBI. Besides, administration of rapamycin improved neurobehavioral function, increased neuronal survival, reduced inflammation and gliosis in injured brain. Therefore, they concluded that rapamycin was neuroprotective following TBI by activation of autophagy. In another study, Zhang et al. (2008) demonstrated that within 1 day after TBI, there were few caspase-3 (+)/LC3 (+) overlapped cells. Whereas after d, the number of caspase-3 (+)/LC3 (+) overlapped cells significantly increased, indicating that autophagy was activated in apoptotic cells after TBI. Furthermore, the protective role of autophagy in TBI was also proposed by Sarkar et al. (2014). Results of their study indicated that impaired autophagy flux was involved in cell death and apoptosis following TBI. Additionally, many neuroprotective drugs have been suggested to attenuate TBI-induced secondary brain injury via activation of autophagy (Xu et al., 2014; Ding et al., 2015; Lin et al., 2016; Gao et al., 2017; Zhang et al., 2017).

### The Detrimental Role of Autophagy in TBI

Support for the detrimental role of autophagy in TBI was initially according to the study conducted by Lai et al. (2008) Oxidative stress could induce autophagy after TBI (Scherz-Shouval et al., 2007), so they used an antioxidant, γ-glutamylcysteinyl ethyl ester (GCEE). Treatment of GCEE alleviated TBI-induced brain tissue loss and neuron death, increased antioxidant reserves, improved Morris-water maze performance and suppressed autophagy formation. Consequently, they speculated that autophagy played a detrimental role in TBI. Moreover, bafilomycin A1 (BafA1) and 3-methyladenine (3-MA), two autophagy inhibitors, were also used. Inhibition of autophagy by BafA1 or 3-MA attenuated behavioral outcome, reduced cell injury, lesion volume and apoptosis after TBI, supporting that autophagy was detrimental for TBI (Luo et al., 2011). Besides, it has been shown that treatment of ketamine could prevent TBI-induced inflammation and exert beneficial effects on memory and behavior by down-regulating Beclin-1 and LC3, suggesting that suppression of autophagy might be a potential therapy to attenuate functional deficits for TBI (Wang C. Q. et al., 2017). Consistent with these conclusions, there were also numerous studies confirming the detrimental role of autophagy in TBI (Feng et al., 2017a; Jiang et al., 2017; Liu et al., 2017; Shen et al., 2017; Tang et al., 2017).

## The Regulation Molecules of Autophagy in TBI

**Figure 2:**
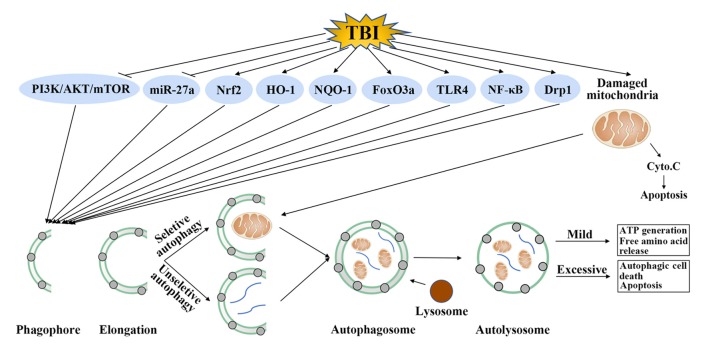
Possible autophagy signaling pathways in traumatic brain injury (TBI). TBI could inhibit phosphatidylinositide 3-kinases (PI3K)/protein kinase B (AKT)/mammalian target of rapamycin (mTOR) pathway and microRNA-27a (miR-27a), activate nuclear factor erythroid 2-related factor 2 (Nrf2), heme oxygenase-1 (HO-1), nicotinamide adenine dinucleotide phosphate, quinine oxidoreductase-1 (NQO-1), forkhead box O 3a (FoxO3a), toll-like receptor 4 (TLR4), nuclear factor kappa-light-chain-enhancer of activated B cells (NF-κB) and dynamin-related protein 1 (Drp1). Regulation of these molecules by TBI further promotes the formation of autophagosome. This step requires unselective or selective targets, such as damaged mitochondria, for degradation. Mild autophagy leads to adenosine triphosphate (ATP) generation and free amino acid release, which are beneficial for TBI. Conversely, excessive autophagy results in autophagic cell death or apoptosis.

Although the accurate role of autophagy in TBI was confused, one undisputable fact was that autophagy was activated after TBI and regulation of autophagy could provide neuroprotection by improvement of cognitive function, decrease of brain edema, protection of blood-brain barrier (BBB) function and suppression of apoptosis, inflammation and oxidative stress (Table 1). The detailed mechanisms mediating the activation of autophagy after TBI is unclear, some regulatory molecules have been suggested which may explain its activation in TBI. It has been shown that TBI could inhibit the phosphatidylinositide 3-kinases/protein kinase B/mammalian target of rapamycin (PI3K/AKT/mTOR) pathway, activate forkhead box O 3a (FoxO3a), dynamin-related protein 1 (Drp1), nuclear factor erythroid 2-related factor 2/antioxidant response element (Nrf2/ARE) pathway and toll-like receptor 4 (TLR4)/nuclear factor kappa-light-chain-enhancer of activated B cells (TLR4/NF-κB) pathway. In addition, these molecules were in the upstream of autophagy and regulation of these molecules by TBI could promote the formation of autophagosome (Figure 2).

**Table 1:** Mechanisms of regulation of autophagy in TBI.

Mechanisms	Factors	Associated molecules	References
Improve cognitive function	Reduce neuronal loss in the hippocampus and cortex	/	Feng et al. (2016)
Attenuate brain edema	Inhibit permeability of endothelial cells	/	Bao et al. (2015) and Tang et al. (2017)
Preserve BBB function	Reduce endothelial cell markers and tight junction protein loss	/	Xu et al. (2014)
Suppress oxidative stress	Interact with Nrf2-ARE pathway and up-regulate the antioxidant enzyme superoxide dismutase activity	Nrf2, HO-1, NQO-1	Zhang et al. (2017)
Reduce apoptosis	Reduce cellular blebbing, chromosomal DNA fragmentation and formation of apoptotic bodies	PI3K/AKT/mTOR, FoxO3a, Drp1	Shen et al. (2017); Sun et al. (2017); Wu et al. (2018)
Inhibit inflammation	Decrease inflammatory factors and attenuate inflammatory response	TLR4, NF-κB	Feng et al. (2016, 2017a,b) and Jiang et al. (2017)

*TBI, traumatic brain injury; BBB, blood-brain barrier; Nrf2, NF-E2-related factor 2; ARE, antioxidant response element; HO-1, heme oxygenase-1; NQO-1, NADPH:quinine oxidoreductase-1; DNA, deoxyribonucleic acid; PI3K/AKT/mTOR, phosphatidylinositide 3-kinases/protein kinase B/mammalian target of rapamycin; FoxO3a, forkhead box O 3a; Drp1, dynamin-related protein 1; TLR4, toll-like receptor 4; NF-κB, Nuclear factor kappa-light-chain-enhancer of activated B cells*.

### PI3K/AKT/mTOR

PI3K/AKT/mTOR signaling pathway is a crucial intracellular pathway in regulation of metabolism, inflammation, cell growth and survival (Huang T. et al., 2018). In addition, this pathway is imbalanced at the occurrence of brain injury (Li et al., 2016). Researches have offered compelling evidence to demonstrate that activation of the PI3K/AKT/mTOR pathway could reduce apoptosis by regulating autophagy in brain injury models (Huang et al., 2017; Lv et al., 2017), including TBI. It has been shown that TBI significantly increased the neurological injury, brain water content, neuron apoptosis and expression of Beclin-1 and LC3-II, while decreased the expression of Phosphorylated (p)-PI3K, p-AKT and p-mTOR in rats. Treatment with dexmedetomidine, a neuroprotective agent, attenuated brain injury, reduced the expression of Beclin-1 and LC3-II, and elevated the expression of the p-PI3K, p-AKT and p-mTOR. Whereas treatment with LY294002, a PI3K/AKT/mTOR pathway inhibitor, observed the opposite trends, indicating that activation of the PI3K/AKT/mTOR pathway provided neuroprotection in TBI via inhibition of autophagy (Shen et al., 2017).

The mechanisms of how PI3K/AKT/mTOR pathway regulates autophagy have been fully explained. mTOR is combined by two mTOR complexes, mTORC1 and mTORC2. mTORC1 (composed of mTOR, Raptor, GLβ and other proteins) is a key negative modulator of autophagy (Cuyàs et al., 2014) and PI3K/AKT pathway is a major regulator of mTORC1 (Manning and Cantley, 2007). AKT activates mTOR by phosphorylation of TSC2 at serine residue 939 (Nellist et al., 2002). Phosphorylated TSC2 subsequently results in the activation of Rheb and promotes mTOR activity (Long et al., 2005; Miyazaki et al., 2010). Therefore, inhibition of AKT suppresses mTOR activity, leads to dephosphorylation of autophagy and Beclin-1 regulator 1 (AMBRA1), activation of unc-51 like autophagy activating kinase 1/2 (ULK1/2) complex, phosphorylation of focal adhesion kinase family interacting protein 200 (FIP200) and finally initiation of autophagy (Wojcik, 2013).

### Nrf2

Nrf2 is a basic leucine zipper redox-sensitive transcription factor that regulates the redox state of cell in harmful stresses (Villeneuve et al., 2010). Under normal conditions, Nrf2 is retained in the cytoplasm by Kelch-like ECH-associated protein 1 (Keap1; Kobayashi et al., 2004). However, under harmful conditions such as oxidative stress, Nrf2 dissociates from Keap1, translocates to the nucleus and activates numerous antioxidant enzymes such as malondialdehyde (MDA), glutathione peroxidase (GPx), heme oxygenase-1 (HO-1) and nicotinamide adenine dinucleotide phosphate, NQO-1 by binding to ARE (de Vries et al., 2008). To date, Nrf2 has been confirmed to provide neuroprotection in various central nervous system (CNS) diseases (Wang et al., 2007; Chen et al., 2011), including TBI (Yan et al., 2008).

There were also studies showing that Nrf2 could regulate autophagy (Li L. et al., 2015; Pajares et al., 2016). The regulation of autophagy by Nrf2 in TBI has been suggested by Zhang et al. (2017) They proposed that the Nrf2-autophagy pathway was activated after TBI to provide neuroprotection both *in vivo* and *in vitro*. However, Nrf2 failed to activate autophagy in Nrf2^−/–^ mice after TBI. Moreover, they found that fucoxanthin, a marine carotenoid extraction from seaweeds, could alleviate TBI-induced brain injury by activation of the Nrf2-autophagy pathway.

But how Nrf2 regulated autophagy has not been fully understood. There were several explanations and these explanations were consistently associated with p62. p62 possess dual-binding sites for ubiquitin chains and LC3. It bound to ubiquitin chains via an ubiquitin-associated (UBA) domain and LC3 through an LC3-interacting region (LIR), leading to the initiation of autophagy (Noda et al., 2008). Moreover, another study indicated that Keap1 uncoupled from Nrf2 could bind to p62, interact with LC3 and transport the ubiquitin conjugate to autophagosome for degradation (Fan et al., 2010). The detailed mechanism of how Nrf2 regulated autophagy was unclear, further studies were needed to clarify it.

### FoxO3a

FoxO3a belongs to the fork frame transcription factor family. It can regulate muscle atrophy, glucose metabolism and apoptosis in cells (Chaanine et al., 2016). Moreover, FoxO3a is expressed in the brain such as the cerebral cortex, hippocampus and cerebellum (Hoekman et al., 2006). Recent studies have revealed that FoxO3a participated in the damage of brain. It has been suggested that FoxO3a was involved in cerebral ischemia and promoted stroke, hence inhibition of FoxO3a could provide neuroprotection against ischemic injury (Yoo et al., 2012; Li D. et al., 2015). Furthermore, FoxO3a was implied in neuronal apoptosis in a rat subarachnoid hemorrhage (SAH) model (An et al., 2015). In addition, FoxO3a has also been confirmed to regulate autophagy. Activated FoxO3a could induce autophagy by directly increasing the transcription of Atgs, such as Beclin-1, LC3, Atg5 and Atg7 (Liu et al., 2015). Besides, FoxO3a could also avtivate autophagy indirectly. Phosphorylation of FoxO3a by AMP-activated protein kinase (AMPK) may repress transcription of S phase kinase-associated protein 2 (SKP2), which subsequently initiated autophagy formation (Sanchez et al., 2012).

FoxO3a also facilitated autophagy to decrease secondary injury after TBI. Knockdown of FoxO3a by small interfering ribonucleic acid (siRNA) significantly inhibited TBI-induced autophagy, thus reversing neuronal damage in the hippocampus and improving neurobehavioral dysfunctions. Whereas activation of autophagy showed the opposite effects (Sun et al., 2018).

FoxOs are a family of proteins that have been found to regulate various cellular functions (Zhou et al., 2012). Besides FoxO3a, other isoforms of FoxO, such as FoxO1, FoxO4 and FoxO6 also express in mammalian cells (Wang et al., 2014). Although they share overlapping structure and function, each member appears to have different tissue-dependent expression patterns and exert a specific biological role. In addition to FoxO3a, other isoforms of FoxO were also showed to regulate autophagy. For example, FoxO1 has been reported to mediate putative kinase 1 (PINK1) transcription and promote autophagy in response to mitochondrial oxidative stress in murine cardiomyocytes (Li W. et al., 2017). However, no reports so far have studied the effects of other FoxO isoforms except FoxO3a on autophagy in TBI models. Therefore, this is an interesting aspect worth exploring.

### TLR4

TLR4 is the first reported mammalian TLR, which has been considered to play an crucial role in initiating the inflammatory reactions and ultimately resulting in neurological defcits in CNS (Wang C. et al., 2012; Fang et al., 2013). For example, the expression TLR4 significantly increased in brain after ICH, and knockout of TLR4 signifcantly ameliorated ICH-induced neurological impairments, cerebral edema and infammatory cytokines expression (Lin et al., 2012). Furthermore, the expression of TLR4 was up-regulated in transient cerebral ischemia, and TLR4-defcient decreased infarct volumes, improved neurobehavioral function and suppressed inflammation after ischemic brain injury (Hyakkoku et al., 2010). The mechanism of how TLR4 regulates inflammation attributes to its initiation of two parallel signaling pathways, the myeloid differentiation primary-response protein 88 (Myd88)/NF-κB pathway and the toll receptor associated activator of interferon (TRIF) pathway. These two signaling pathways subsequently activate transcription factors that regulate proinflammatory cytokine genes (Buchanan et al., 2010).

TLR4 has also been suggested to regulate autophagy in brain injury models such as TBI. Jiang et al. (2017) found that knockdown of TLR4 ameliorated neuroinfammatory response after TBI by inhibition of autophagy. In addition, many drugs such as resveratrol, resatorvid and apocynin have been reported to provide neuroprotection in TBI by inhibiting the TLR4-mediated autophagy pathway (Feng et al., 2016, 2017a,b). But how TLR4 enhanced autophagy remained unclear. An NF-κB binding site has been found in the promoter region of Beclin-1 genes. Therefore, activation of NF-κB by TLR4 may up-regulate Beclin-1 expression and promote autophagy (Copetti et al., 2009).

TLRs are a group of pattern recognition receptors present in cytoplasm and cell membrane, and can specifically recognize pathogen-associated molecular patterns. Interestingly, in a white matter injury (WMI) model, TLR3 was colocalized with the ER and autophagosome in ventral lateral posterior neurons, indicating that autophagy could be regulated by TLR3 (Vontell et al., 2015). Since TLR3 also participated in the process of inflammation (Liu et al., 2018), so whether inhibiton of TLR3 could suppress TBI-induced inflammation by modulation of autophagy was unclear, which required further researches.

### Drp1

Drp1 is a dynamin-like GTPase shuttling between the mitochondrial and cytoplasm surface and it mediates mitochondrial fission by calcium-dependent dephosphorylation (Smirnova et al., 2001). Drp1 is highly expressed in brain neurons and has been investigated in Alzheimer’s disease, Parkinson’s diseases (PDs), stroke, epilepsy and TBI (Knott and Bossy-Wetzel, 2008; Qiu et al., 2013; Zuo et al., 2014; Kim et al., 2016). Recent researches showed that inhibition of Drp1 could decrease brain injury and apoptosis after TBI by maintaining mitochondrial functions (Wu et al., 2016).

Drp1 is also a crucial upstream protein of autophagy. When Drp1 is stimulated by reactive oxygen species (ROS) or damaged mitochondrial deoxyribonucleic acid (DNA), mitochondrion becomes depolarized and damage. The damaged mitochondrion is then recognized by autolysosome for degradation, this process is named mitophagy (Song et al., 2015). It has been suggested that Drp1 not only mediated BCL2/adenovirus E1B 19 kilodalton interacting protein-3 (BNIP3)-induced mitophagy in adult cardiomyocytes (Tanaka et al., 2010) but also participated in Parkin-induced mitophagy in mouse embryonic fibroblast (MEF) cells (Lee et al., 2011). Consistent with these results, one study demonstrated that suppression of Drp1 alleviated TBI-induced BBB disruption and apoptosis by inhibiting mitophagy.

## Other Upstream Molecules of Autophagy Worth Studying in TBI

TBI is a complex disease involving many pathological processes. There are several molecular targets responsible for the secondary damage of TBI. Although the effects of molecules such as Nrf2 on autophagy have been widely described in TBI, the effects of other molecules such as long noncoding RNA (LncRNA) and BNIP3 on autophagy in TBI have not been fully explained so far.

### LncRNA

LncRNA is an RNA molecule that is longer than 200 nucleotides and is not translated to a protein (Spizzo et al., 2012). LncRNAs were primarily regarded as transcriptional by-products. However, there was considerable evidence indicating that lncRNAs were invloved in many pathological processes (Batista and Chang, 2013). Indeed, the role of lncRNAs in TBI was definite. The expression of LncRNAs was significant changed in the injury brain after TBI (Zhong et al., 2016; Wang C. F. et al., 2017). Knockdown or overexpression of lncRNAs could suppress TBI-induced inflammation and apoptosis, resulting in better outcome in mice (Yu et al., 2017; Zhong et al., 2017).

Additionly, lncRNAs was emerging as new factors involved in autophagy in brain injury models. It has been revealed that down-regulation of metastasis-associated lung adenocarcinoma transcript 1 (MALAT1) attenuated neuronal cell death by suppressing autophagy in cerebral ischemic stroke (Guo et al., 2017). Moreover, MALAT1 played a protective role against oxygen-glucose deprivation/reoxygenation-induced injury in brain microvascular endothelial cell (BMEC) by enhancing autophagy (Li Z. et al., 2017). Furthermore, lncRNA nuclear paraspeckle assembly transcript 1 (NEAT1) stabilized phosphatase and tensin homolog (PTEN)-induced PINK1 protein by promoting 1-methyl-4-phenyl-1,2,3,6-tetrahydropyridine (MPTP)-induced autophagy in PD (Yan et al., 2018). Thus, it can be speculated that lncRNAs could also regulate autophagy in TBI. Further studies are needed to clarify it.

### BNIP3

BNIP3 belongs to the unique family of death-inducing mitochondrial proteins (Chen et al., 1997). Under hypoxic conditions, BNIP3 is activated by transcriptional factor hypoxia inducible factor 1 (HIF-1) and promotes cell survival (Swiderek et al., 2013). BNIP3 has also been studied in TBI. It has been shown that inhibiton of BNIP3 by 2-methoxyestradiol (2ME2) could provide neuroprotection after TBI by inhibiton of secondary brain damage such as apoptosis and oxidative stress (Schaible et al., 2014).

The role of BNIP3 in autophagy is well established (Xin et al., 2011; Lu et al., 2016). BNIP3 can suppress mTOR by binding to and inhibiting Rheb, and subsequently induce autophagy (Zhang and Ney, 2011). Furthermore, BNIP3 can trigger the dissociation of Beclin-1 and B-cell lymphoma 2 (Bcl-2) by competing with Beclin-1 for binding to Bcl-2 to activate autophagy (Glick et al., 2010). So, it is necessary to examine whether BNIP3 could regulate autophagy in TBI in future studies.

## Related Therapeutics Agents Targeting Autophagy in TBI

Numerous proof-of-principle studies have indicated that regulation of autophagy by pharmacological activators or inhibitors could attenuate TBI-induced brain injury in preclinical studies and represent a promising therapeutic approach for TBI. Therefore, we elucidate the autophagy activators and inhibitors used in TBI in this section (Table 2).

**Table 2:** Summary of therapeutics development targeting autophagy in TBI.

Methods or Compounds	Initial research time	Effects on autophagy	Doses	Functions in TBI	References
Luteolin	2014	Activate	30 mg/kg	Reduced neuronal degeneration, alleviated brain edema and BBB disruption, inhibited inflammatory response	Xu et al. (2014)
Melatonin	2015	Activate	10 mg/kg	Improved neurological deficits, decreased brain edema and apoptosis	Ding et al. (2015)
Moderate hypothermia	2015	Activate	/	Decreased cell death	Jin et al., 2015
17AAG	2015	Activate	24 mg	Attenuate brain edema, neuronal death and apoptosis, improved the recovery of motor function.	Ma et al. (2015)
FTY720	2016	Activate	0.5 mg/kg	Improved neurobehavioral function, alleviated brain edema and apoptosis	Zhang et al. (2016)
LY294002	2016	Activate	10 μmol/L	Increased neurological injury and brain water content	Zhang et al. (2016)
Methylene blue	2016	Activate	1 mg/kg	Ameliorated neurological functional deficits, inhibited cerebral lesion volumes, brain edema and microglial activation	Zhao et al. (2016)
THC	2017	Activate	5 mg/kg	Improved neurological function, reduced the brain water content, oxidative stress and apoptosis	Gao et al. (2017)
Fucoxanthin	2017	Activate	100 mg/kg, 0.05 mmol/L	Improved neurological deficits, decreased cerebral edema, brain lesion, neuronal apoptosis and oxidative stress	Zhang et al. (2017)
3-MA	2011	Suppress	400 nmol/L	Improved behavioral outcome, reduced cell apoptosis and lesion volume	Luo et al. (2011)
BafA1	2011	Suppress	4 nmol/L	Improved behavioral outcome, reduced cell apoptosis and lesion volume	Luo et al. (2011)
Necrostatin-1	2012	Suppress	2.6 μg	Reduce tissue damage, functional deficits and apoptosis	Wang et al. (2012)
Humanin	2013	Suppress	0.1 μg	Improved motor performance, reduced lesion volume and apoptosis	Wang et al. (2013)
Resveratrol	2014	Suppress	100 mg/kg, 5 μmol/L	Attenuated brain edema, improved spatial cognitive function and neurological impairment, decreased apoptosis and inflammation	Lin et al. (2014)
Ceftriaxone	2014	Suppress	200 mg/kg	Attenuated brain edema and cognitive function deficits	Cui et al. (2014)
Hydrogen sulfide	2014	Suppress	1 μmol/kg	Ameliorated motor performance, reduced brain edema and apoptosis	Zhang et al. (2014)
Apelin-13	2015	Suppress	0.05 mg	Attenuated neural cell death, lesion volume and neural dysfunction	Bao et al. (2015)
Chloroquine	2015	Suppress	3 mg/kg	Reduced cerebral edema and motor and cognitive functional deficits, suppressed inflammation	Cui et al. (2015)
Rosiglitazone	2015	Suppress	2 mg/kg	Reduced neuronal apoptosis and inflammation, increased functional recovery	Yao et al. (2015)
Quercetin	2016	Suppress	50 mg/kg	Improved cognitive function and neurological impairment, attenuated apoptosis	Du et al. (2016)
Ketamine	2017	Suppress	10 mg/kg	Ameliorated behavioral and histopathological outcomes, exerted anti-inflammatory effects, increased ATP content	Wang C. Q. et al. (2017)
Overexpress of miR-27a	2017	Suppress	/	Attenuated neurological deficits and brain injury	Sun et al. (2017)
Calcitriol	2017	Suppress	1 μg/kg	Attenuated neurological deficits and apoptosis	Cui et al. (2017)
Apocynin	2017	Suppress	50 mg/kg	Ameliorated motor and behavioral impairment, brain edema, neuronal damage and inflammation	Feng et al. (2017a)
Knockdown of TLR4	2017	Suppress	/	Improved neurological deficits, reduced brain edema and neuronal damage, ameliorated neuroinflammatory response	Jiang et al. (2017)
Resatorvid	2017	Suppress	0.5 mg/kg	Attenuated neurons loss, brain edema, neurobehavioral impairment neuroinflammation responses	Feng et al. (2017b)
Dex	2017	Suppress	15 μg/kg	Reduced cerebral edema and inflammatory reaction	Shen et al. (2017)
FGF2	2017	Suppress	250 μg/kg	Alleviated brain edema, reduced neurological deficits, prevented tissue loss and increased the number of surviving neurons	Tang et al. (2017)
DHA	2018	Suppress	16 mg/kg	Reduced hippocampal damage and white matter injury, improved neurological function	Yin et al. (2018)
Knockdown of FoxO3a	2018	Suppress	/	Improved neurobehavioral dysfunction, reversed neuronal damage	Sun et al. (2018)
Mdivi-1	2018	Suppress	3 mg/kg	Attenuated blood-brain barrier disruption and cell death	Wu et al. (2018)
Pifithrin-α	2018	Suppress	2 mg/kg	Improved motor deficits, suppressed striatal glial activation, inflammation, apoptosis and oxidative damage	Huang Y.-N. et al. (2018)

*TBI, traumatic brain injury; BBB, blood-brain barrier; 17AAG, 17-allylamino-demethoxygeldanamycin; THC, tetrahydrocurcumin; 3-MA, 3-methyladenine; BafA1, bafilomycin A1; ATP, adenosine triphosphate; miR-27a, microRNA-27a; TLR4, toll-like receptor 4; Dex, dexmedetomidine; FGF2, fibroblast growth factor-2; DHA, docosahexaenoic acid; FoxO3a, Forkhead box O 3a; Mdivi-1, mitochondrial division inhibitor 1*.

### Autophay Inducers

#### Luteolin

Luteolin was shown to activate autophagy in TBI (Xu et al., 2014). Luteolin is a member of the flavonoid family and is abundant in vegetables and fruits such as broccoli, parsley and celery (Mencherini et al., 2007). Luteolin has a variety of pharmacological properties such as antioxidant, anti-inflammation and cancer preventive effects (Neuhouser, 2004). Besides, luteolin was reported to regulate autophagy in multiple models including TBI (Zhang B. C. et al., 2016; Cao et al., 2017). It has been shown that autophagy was protective after TBI and treatment of luteolin further enhanced autophagy by activating NF-κB, leading to decreased brain injury, inflammation and apoptosis following TBI (Xu et al., 2014).

#### Melatonin

Melatonin (N-acetyl 5-methoxytryptamine) was reported to activate autophagy in TBI (Ding et al., 2015). It is an autocrine hormone mainly produced by the pineal gland and regulates the sleepe cycle (Brzezinski, 1997). Melatonin can cross the BBB easily to provide neuroprotection (Cheung et al., 2006). Melatonin has been recognized as a powerful antioxidant that protect antioxidative enzymes from oxidative damage (Galano et al., 2013). In addition, studies have shown that melatonin activated autophagy in many models inclduing TBI (Chen et al., 2014; Kucharewicz et al., 2018; Pan et al., 2018). Activation of autophagy by melatonin was found to provide neuroprotection in TBI by suppression of inflammation and oxidative stress, suggesting that autophagy was beneficial for TBI (Ding et al., 2015).

#### Fucoxanthin

In 2017, fucoxanthin was proposed to exhibit neuroprotective effects after TBI by activation of autophagy (Zhang et al., 2017). Fucoxanthin is the most abundant marine carotenoid extraction in seaweeds and is considered as a powerful antioxidant (Sugawara et al., 2002). It has been proposed to exhibit a variety of pharmacological properties such as inhibiting tumor growth, repressing inflammation reaction and reducing oxidative stress by activation of autophagy (Hou et al., 2013; Moskalev et al., 2018). Furthermore, activation of autophagy was shown to provide neuroprotection after TBI and administration of fucoxanthin post-TBI could attenuated TBI-induced neurological defcits, cerebral edema, brain lesion, neuronal apoptosis and oxidative stress by further promoting autophagy (Zhang et al., 2017).

#### Tetrahydrocurcumin (THC)

Tetrahydrocurcumin (THC) is extracted from the roots of the Curcuma longa Linn. It owns antioxidant and anti-inflammatory activity *in vitro* and *in vivo* (Wu et al., 2014). Besides, THC could protect cerebral ischemia and neurodegenerative diseases against oxidative stress by modulation of autophagy (Mishra et al., 2011; Tyagi et al., 2012). Furthermore, the effects of THC on autophagy after TBI has also been investigated in 2017. Gao et al. (2017) found that THC improved neurological function, ameliorated cerebral edema, reduced oxidative stress and decreased the number of apoptotic neurons by activation of autophagy in a rat model of TBI, confirming the protective role of autophagy in autophagy.

### Autopahgy Inhibitors

#### Necrostatin-1 (NEC-1)

As a special receptor-interacting protein-1 (RIP-1) inhibitor to depress necroptotic cell death, Necrostatin-1 (NEC-1) has been a hot topic of therapeutic agent in different models (Degterev et al., 2008). NEC-1 has been shown to improve functional outcomes and reduce the disrupture of brain tissue in TBI models (You et al., 2008). Moreover, previous studies have indicated that necroptosis was closely associated with autophagy and apoptosis, and thereby, suppression of necroptosis by NEC-1 may interfere with the process of autophagy and apoptosis. Rosenbaum et al. (2010) found that NEC-1 could decrease the expression of LC3-II after retinal ischemic. Furthermore, NEC-1 was found to inhibit autophagy in TBI in 2012. Wang Y. Q. et al. (2012) proposed that activation of autophagy could increase apoptosis after TBI and treatment of NEC-1 suppressed TBI-induced autophagy, leading to decreased apoptosis. These results indicated that autophagy played a detrimental role in TBI.

#### Apelin-13

Apelin-13 is the endogenous ligand of the APJ receptor. It is extracted from bovine stomachs (Tatemoto et al., 1998). Previous studies have shown that apelin-13 could attenuate postischemic cerebral edema and brain injury by suppressing apoptosis (Khaksari et al., 2012). Besides, apelin-13 could suppress glucose deprivation-induced cardiomyocyte autophagy (Jiao et al., 2013). The effects of apelin-13 on autophagy in TBI has also been confirmed in 2014. Bao et al. (2015) suggested that autophagy was activated and lead to secondary brain damage such as apoptosis after TBI. Adminstration of apelin-13 could reverse TBI-induced secondary brain damage by inhibiting autophagy.

#### Ketamine

Ketamine is usually used for starting and maintaining anesthesia (Green et al., 2011). Other functions of ketamine include sedation and acesodyne in intensive care (Zgaia et al., 2015). In addition to these effects, ketamine has been shown to provide neuroprotection for TBI patients by decreasing glutamate excitotoxicity and inflammatory factors (Chang et al., 2009; Bhutta et al., 2012). Moreover, in 2017, one study showed that autophagy promoted apoptosis and inflammation after TBI while treatment of ketamine could decrease autophagy by activation of the mTOR signaling pathway, thus ameliorating apoptosis and inflammation in TBI (Wang C. Q. et al., 2017).

#### Docosahexaenoic Acid (DHA)

Docosahexaenoic acid (DHA) is an omega-3 fatty acid that is a primary structural component of human brain. It can be extracted from fish oil and milk or synthesized by alpha-linolenic acid (Guesnet and Alessandri, 2011). DHA has been shown to provide neuroprotection by improving neurological deficits, decreasing infarct volume and reducing proapoptotic proteins (Belayev et al., 2009; Mayurasakorn et al., 2011). Furthermore, Yin et al. (2018) found that TBI significantly elevated the ATG preteins such as sequestosome 1 (SQSTM1/p62), lysosomal-associated membrane proteins 1 (Lamp1), Lamp2 and cathepsin D (Ctsd) in the rat hippocampusm, which led to decreased cognitive functions as well as both gray matter and white matter damages in rats. However, DHA treatment suppressed TBI-induced autophagy and reversed the hippocampal lysosomal biogenesis and function, suggesting that autophagy was detrimental for TBI and suppression of autophagy exhibited neuroprotective effects after TBI.

### Other Autophagy Regulators

Recently, there were some other autophagy activators or inhibitors that have been proposed in TBI models such as pifithrin-α (PFT-α; Huang Y.-N. et al., 2018), apocynin (Feng et al., 2017a), trehalose (Portbury et al., 2017), dexmedetomidine (Shen et al., 2017), mitochondrial division inhibitor 1 (Mdivi-1; Wu et al., 2018) and so on (Wang et al., 2013; Cui et al., 2014, 2015, 2017; Lin et al., 2014; Zhang et al., 2014; Jin et al., 2015; Ma et al., 2015; Yao et al., 2015; Du et al., 2016; Zhang L. et al., 2016; Zhao et al., 2016; Sun et al., 2017). All these agents exerted neuroprotective effects in models of TBI, possibly by activation or inhibition of autophagy.

## Possible Reasons for the Dual Role of Autophagy in TBI

The mixed results of these studies may be due to the activation degree of autophagy in TBI. Mild autophagy could lead to adenosine triphosphate (ATP) generation, which is beneficial for cell survival. Conversely, excessive autophagy may promote autophagic cell death or apoptosis (Hakumäki et al., 1999). Depending on different environment and stimulus of brain trauma, the activation degree of autophagy may also be different. Therefore, activation of mild autophagy or suppression of excessive autophagy could be both benefit for TBI.

In addition, the available studies exploring the role of autophagy in TBI relied on non-selective drugs that affected autophagy. Therefore, we must consider the effects of these drugs on other signaling pathways instead of the simple influence of autophagy. For example, 3-MA is a non-specific PI3K inhibitor, which may also regulate other pathways such as inflammation and apoptosis. As mentioned above, 3-MA was shown to decrease neuron cell death and improve neurological function after TBI. However, it was difficult to determine whether the protective role of 3-MA was due to its effects on inhibiting autophagy or inflammation or other pathways. Therefore, these studies drew diametrically opposing viewpoints as to the role of autophagy in TBI. The development of specific agents that regulated autophagy may help to clearly elucidate the role of autophagy in TBI. Meanwhile, with the help of molecular biology technology, the precise knockdown or knockout of ATG genes can be realized. Knockdown of the gene for encoding Beclin-1 protein has been achieved in a cerebral ischemia model (Xing et al., 2012). These are all directions of future researches.

## Concluding Remarks

Autophagy plays an important role in TBI, it participates in a variety of cellular and molecular processes of TBI. In this review article, we describe the mechanism of autophagy, the functions of autophagy in TBI as well as some upstream moleculars and pharmacological regulators of autophagy involved in TBI. These observations make autophagy an attractive therapeutic target for developing new therapeutic strategies to achieve better outcomes for patients suffering from TBI.

## Author Contributions

LZ wrote this review article. HW edited and revised it.

## Conflict of Interest Statement

The authors declare that the research was conducted in the absence of any commercial or financial relationships that could be construed as a potential conflict of interest.
